# Celastrol Enhanced Doxorubicin‐Mediated Apoptosis in Saos‐2 Osteosarcoma Cells

**DOI:** 10.1155/bmri/8836764

**Published:** 2026-05-18

**Authors:** Fatemeh Pilevar, Dariush Shanehbandi, Amir Valizadeh, Leila Alem, Mehran Molavand, Maryam Majidinia, Bahman Yousefi, Elahe Mohammadian

**Affiliations:** ^1^ Department of Biochemistry, Faculty of Medicine, Tabriz University of Medical Sciences, Tabriz, Iran, tbzmed.ac.ir; ^2^ Immunology Research Center, Tabriz University of Medical Sciences, Tabriz, Iran, tbzmed.ac.ir; ^3^ Student Research Committee, Tabriz University of Medical Sciences, Tabriz, Iran, tbzmed.ac.ir; ^4^ Solid Tumor Research Center, Urmia University of Medical Sciences, Urmia, Iran, umsu.ac.ir; ^5^ Molecular Medicine Research Center, Tabriz University of Medical Sciences, Tabriz, Iran, tbzmed.ac.ir; ^6^ Health Science Faculty, Nursing Department, Bahçeşehir Cyprus University Alayköy Campus, Mersin 10, Turkey

**Keywords:** apoptosis, celastrol, doxorubicin, osteosarcoma

## Abstract

**Background:**

Osteosarcoma is one of the most lethal types of human malignancy in the world. Natural compounds found in certain plant foods are considered chemotherapeutic agents that exert their effects through antiproliferative, proapoptotic, and pro‐oxidant properties associated with cancer.

**Objective:**

In this study, we investigated the effects of celastrol on the doxorubicin‐mediated apoptosis in osteosarcoma cells. Saso‐2 cancer cells were treated with ascending concentrations of doxorubicin and celastrol alone or in combination.

**Methods:**

Cytotoxicity was measured using the MTT assay. Flow cytometry with Annexin V‐FITC/PI double staining was performed to quantify apoptotic cell populations. Western blot and quantitative reverse transcription‐polymerase chain reaction (qRT‐PCR) were used to measure the protein and mRNA expression levels of apoptosis mediators. An ELISA kit measured apoptosis.

**Result:**

In Saos‐2 cells, celastrol enhanced the cytotoxic effects of doxorubicin. A combination of doxorubicin and celastrol had a more potent effect on apoptosis induction and modulation of mRNA and protein levels of key apoptotic mediators, including Bax, Bcl‐2, caspase‐3, cytochrome c, and p53.

**Conclusion:**

Overall, this study revealed that doxorubicin and celastrol could significantly potentiate doxorubicin‐mediated apoptosis in osteosarcoma cells.

## 1. Introduction

Osteosarcoma is one of the most common types of bone cancer. The prevalence of this tumor is higher in teenagers and young people [1]. In osteosarcoma, most long bones are involved, and the most common sites are the bones above and below the knee [2]. The severity of malignancy in osteosarcoma is very high, and metastases are seen in 80%–90% of patients. Most osteosarcoma metastases occur in the lungs and, to a lesser extent, in the brain, prostate, and kidney [3]. Until the 1970s, the primary treatment was amputation, and the chance of survival for patients within 5 years was only 15%–20%. In recent years, conventional treatments for osteosarcoma have been chemotherapy and surgery, and osteosarcoma does not respond well to radiotherapy [4]. Chemotherapy is often performed before surgery to reduce the size of the tumor, and after surgery, it is suitable to destroy the remaining cells. Over the last 25 years, the 5‐year survival rate has reached 60% [5]. However, because of the resistance of osteosarcoma to chemotherapy drugs, tumor recurrence and side effects caused by common treatments such as surgery and chemotherapy, the use of biological methods such as gene therapy will be a new approach to the treatment of this cancer, for which a wide understanding is needed [6]. It is necessary to understand the molecular mechanisms that promote this cancer, and this issue will significantly help in the diagnosis and treatment of osteosarcoma and increase the chances of survival of patients [7].

Recent decades have witnessed a considerable increase in attention to the application of natural compounds for treating a broad range of cancers due to their low cost, safety, and potent anticancer effects [8]. Among these compounds, celastrol, one of the most abundant and promising bioactive compounds derived from the *Tripterygium wilfordii* Hook F (TWHF) plant, is extensively studied in cancer treatment, with promising findings reported. Several clinical studies conducted on the effects of celastrol show promising results about its anticancer activities against various tumors in breast cancer, leukemia, prostate cancer, melanoma, liver cancer, etc. [9]. In vitro and in vivo investigations have revealed that celastrol‐mediated inhibitory effects on cancer cells are mainly due to its proapoptotic, pro‐autophagic, antiproliferative, anti‐inflammatory, anti‐angiogenic, and anti‐metastatic impacts, as well as modulation of key signaling pathways involved in cancer cell proliferation and apoptosis [10, 11]. In addition, celastrol has been reported to enhance cancer cell sensitivity to conventional chemotherapeutic agents and to reverse drug resistance [12]. However, studies on the efficacy of celastrol in potentiating the anticancer effects of doxorubicin and increasing doxorubicin‐mediated apoptosis are limited [13]. Therefore, this study aimed to investigate the effects of celastrol in enhancing doxorubicin‐mediated apoptosis in osteosarcoma Saos‐2 cells.

## 2. Materials and Methods

### 2.1. Cell Culture

The human osteosarcoma Saos‐2 cell line was obtained from the institute’s cell bank and cultured in RPMI 1640 medium containing 10% fetal bovine serum (FBS). Cell culture was carried out in a humid atmosphere containing 5% carbon dioxide at 37°C.

### 2.2. MTT Assay

The effects of celastrol on doxorubicin‐mediated inhibition of Saos‐2 cell proliferation were investigated using an MTT assay. In this method, culture medium containing Saos‐2 cells (5 x 10^3^ cells per well) was added to the wells of the plates. Then, the culture medium containing different concentrations of celastrol, doxorubicin, and their combination was added to the cells. Some of the wells are treated with drug solvent, and some of the wells without treatment are considered as a negative control. After multiple time points (0, 12, 24, 48, and 72 h) of incubation, culture medium containing MTT was replaced with the previous culture medium and incubated for 4 h. Next, DMSO and Sorenson’s phosphate buffer are added to the wells, and an ELISA plate reader measures absorbance at 570 nm.

### 2.3. qRT‐PCR

The mRNA expression levels of key apoptosis mediators, including Bax, Bcl‐2, Caspase‐3, p53, and cytochrome c, were measured using qRT‐PCR. In this method, we cultured the cells in 6‐well plates at a density of 200,000 cells per well and treated them with celastrol, doxorubicin, and their combination for 48 h. Then, cellular RNA was purified using the RNA extraction kit. Then, cDNA was synthesized using oligo‐dT primers and extracted RNA. Finally, a PCR test is performed using specific primers designed for the desired genes, along with relevant bioinformatics software and a master mix PCR containing SYBR Green dye. Gene expression was calculated using CT values and the 2^−*ΔΔ*ct^ method.

### 2.4. Western Blotting

To perform a Western blot, treated and control cells in 6‐well plates were centrifuged and separated from the supernatant. After washing with PBS, the cell lysate was prepared using lysing reagent (RIPA buffer), and the protein concentration was measured by a biophotometer. (10%) PAGE‐SDS separated proteins in cell lysates and then transferred them to a PVDF membrane. In the next step, the PVDF membrane was blocked, and primary antibodies against Bax, Bcl‐2, Caspase‐3, p53, and cytochrome c, and *β*‐actin proteins were added. After 1 h of incubation with peroxidase‐labeled secondary antibody, the enzyme substrate was added. Protein bands were visualized using an ECL detection system and captured using a gel documentation system. Densitometric quantification of band intensities was performed using ImageJ software (version 1.53, NIH, United States). Protein expression levels were normalized to *β*‐actin and expressed as fold change relative to control. Data represent mean ± SD from three independent experiments.

### 2.5. Measuring Caspase‐3 Activity

Caspase‐3 activity will be measured using the caspase‐3 colorimetric kit. Saos‐2 cells will be cultured in a 6‐well plate with a density of 5000 cells per well for 24 h. Then, the cells were treated with doxorubicin and celastrol. Dithiothreitol and IEDT‐pna (a Cas3 substrate) were used to measure the caspase‐3 activity. Finally, the samples were diluted to 900 *μ*L, and their absorbance was measured at 405 nm.

### 2.6. Measuring Apoptosis

To evaluate the effects of celastrol and doxorubicin, alone or in combination, on apoptosis in Saos‐2 cells, the Cell Death Detection ELISA kit was used. Cell death was evaluated using a commercial ELISA kit according to the manufacturer’s protocol.

### 2.7. Flow Cytometry Analysis of Apoptosis

Apoptosis was quantified using the Annexin V‐FITC/PI double‐staining kit (BioLegend, San Diego, CA, United States) according to the manufacturer’s protocol. Saos‐2 cells were seeded at a density of 2 × 10^5^ cells per well in 6‐well plates and allowed to adhere overnight. Cells were then treated with doxorubicin (10 *μ*M), celastrol (4 *μ*M), or their combination for 48 h. Following treatment, both floating and adherent cells were collected by trypsinization, washed twice with cold PBS, and resuspended in 1× binding buffer at a concentration of 1 × 10^6^ cells/mL. Subsequently, 100 *μ*L of cell suspension (approximately 1 × 10^5^ cells) was transferred to flow cytometry tubes and incubated with 5 *μ*L of Annexin V‐FITC and 5 *μ*L of propidium iodide (PI) for 15 min at room temperature in the dark. After adding 400 *μ*L of 1× binding buffer, samples were immediately analyzed using a BD FACSCalibur flow cytometer (BD Biosciences, San Jose, CA, United States). For each sample, at least 10,000 events were acquired. Data analysis was performed using FlowJo software version 10.8 (Tree Star, Ashland, OR, United States). Cells were classified into four populations: viable cells (Annexin V^−^/PI^−^), early apoptotic cells (Annexin V^+^/PI^−^), late apoptotic cells (Annexin V^+^/PI^+^), and necrotic cells (Annexin V^−^/PI^+^). The total apoptotic rate was calculated as the sum of early and late apoptotic cell percentages.

### 2.8. Statistical Analysis

All the data presented in this study are expressed as mean ± SD from three identical experiments conducted in three replicates. Statistical analyses between groups over time were conducted using analysis of variance (ANOVA), followed by Tukey’s post hoc test for statistical comparisons. *p* values less than 0.05 were considered statistically significant. All statistical analyses were performed using GraphPad Prism version 7.00 (GraphPad, San Diego, CA).

## 3. Results

### 3.1. Celastrol Potentiated Doxorubicin‐Mediated Suppressive Effects on the Viability of Saos‐2 in a Dose‐Dependent Manner

**Figure 1:**
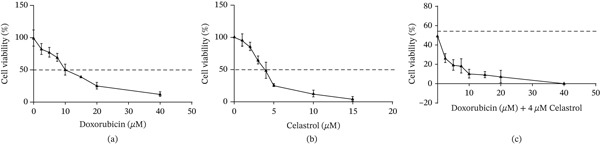
The antiproliferative effects of celastrol and doxorubicin alone or in combination. (a) Dose–response curve of doxorubicin in Saos‐2 cells at 48 h (IC50 = 10 ± 5 *μ*M). (b) Dose–response curve of celastrol in Saos‐2 cells at 48 h (IC50 = 3.95 ± 0.4 *μ*M). (c) Combination treatment with celastrol (4 *μ*M) and various concentrations of doxorubicin at 48 h, demonstrating enhanced cytotoxicity. Data represent mean ± SD from three independent experiments performed in triplicate. *p* < 0.05 vs. control; ^#^ *p* < 0.05 vs. doxorubicin alone.

An MTT assay was performed to measure the cytotoxic effects of doxorubicin after exposure of Saos‐2 cells to increasing concentrations. Upon 48 h incubation, cytotoxicity was measured (Figure 1a). Increasing doxorubicin concentration from 0 to 40 *μ*M increased its inhibitory effect on cell viability, almost completely suppressing it at higher concentrations. In the Saos‐2 cell line, doxorubicin IC50 was 10 ± 5 *μ*M after 48 h of treatment. Celastrol also exerted dose‐dependent antiproliferative effects on Saos‐2 cells after 48 h (Figure 1b). The IC50 value for celastrol in Saos‐2 cells was 3.95 ± 0.4 *μ*M. More importantly, celastrol (4 *μ*M) enhanced the antiproliferative effects of doxorubicin at various concentrations. As represented in Figure 1c, celastrol decreased the IC50 value of doxorubicin from 10 to 4.8 *μ*M in Saos‐2 cells, which indicated the efficacy of celastrol in potentiating doxorubicin‐mediated suppressive effects on the viability of Saos‐2 cells. At a concentration of 4 *μ*M (approximately IC50), celastrol reduced cell viability to 50.2*%* ± 3.8*%*, while the combination treatment reduced viability to 22.5*%* ± 2.9*%*, demonstrating significant synergistic effects (*p* < 0.001).

Time‐course analysis of celastrol‐induced cytotoxicity in Saos‐2 osteosarcoma cells measured by MTT assay at 0, 12, 24, 48, and 72 h. The Saos‐2 cell viability over time: 98.5*%* ± 3.1*%* at 0 h, 89.3*%* ± 3.5*%* at 12 h, 81.2*%* ± 4.2*%* at 24 h, 50.2*%* ± 3.8*%* at 48 h, and 38.7*%* ± 4.5*%* at 72 h (Figure S1). The data demonstrate the time‐dependent reduction in cell viability following treatment with 4 *μ*M celastrol.

### 3.2. Celastrol Upregulated p53 Expression in Saos‐2 Cells Treated With Doxorubicin

**Figure 2:**
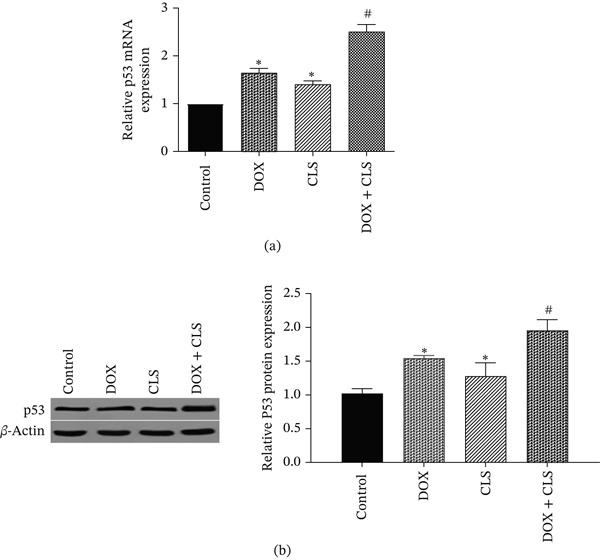
The mRNA and protein expression levels of p53 in Saos‐2 cells treated with celastrol and doxorubicin alone or in combination with each other. Data represent mean ± SD from three independent experiments.  ^∗^ *p* < 0.05 vs. control; ^#^ *p* < 0.05 vs. single treatments.

p53 is among the most important proliferative genes aberrantly expressed in various cancers, including osteosarcoma, and plays a pivotal role in their pathogenesis. Hence, we measured p53 protein and mRNA expression levels in Saos‐2 cells treated with celastrol and doxorubicin, alone or in combination. We found that Saos‐2 cells exposed to 10 *μ*M doxorubicin and 4 *μ*M celastrol showed higher mRNA and protein expression levels of p53, a main tumor suppressor gene, compared with mono‐treatments (*p* < 0.05; Figure 2a,2b).

### 3.3. Celastrol Enhanced Doxorubicin‐Mediated Apoptosis in Saos‐2 Cells

**Figure 3:**
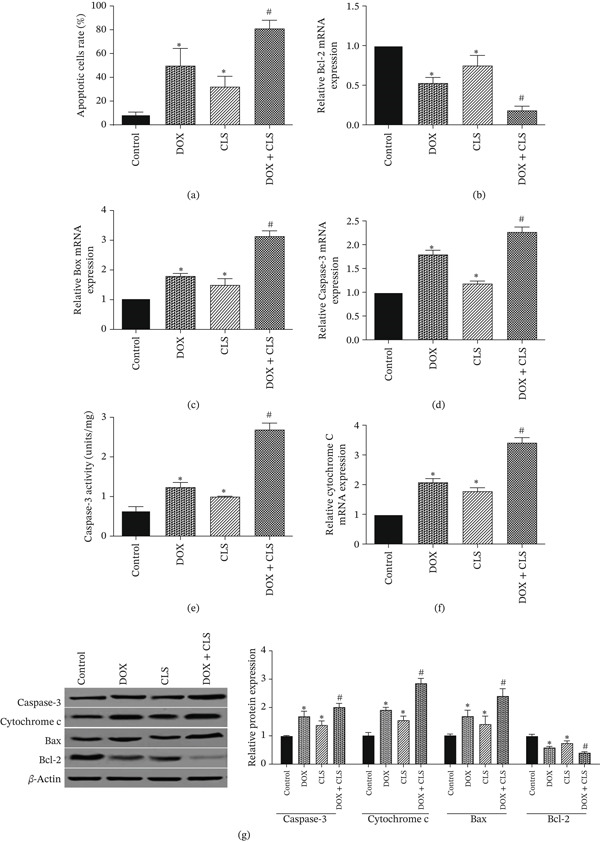
Celastrol enhanced doxorubicin‐mediated apoptosis in Saos‐2 cells. Data represent mean ± SD from three independent experiments.  ^∗^ *p* < 0.05 vs. control; ^#^ *p* < 0.05 vs. single treatments.

ELISA results demonstrated that the percentage of apoptotic cells was significantly higher in Saos‐2 cells treated with a combination of doxorubicin and celastrol than in cells treated with doxorubicin or celastrol alone (*p* < 0.05; Figure 3a). Additionally, ELISA results were approved by qRT‐PCR and western blot, which measured Bax, Bcl‐2, and caspase‐3 levels. As shown in Figure 3, the combination of doxorubicin and celastrol downregulated the expression levels of antiapoptotic genes, including Bcl‐2 (Figure 3b). They upregulated the expression levels of proapoptotic genes, including Bax (Figure 3c) and caspase‐3 (Figure 3d), at both mRNA and protein levels. The caspase‐3 activity (Figure 3e) also showed similar effects, such that a potent increase in caspase‐3 activity was found upon combinational treatment (Figure 3e). The mRNA and protein expression levels of cytochrome c were also observed to be significantly increased in Saos‐2 cells treated with a combination of doxorubicin and celastrol. This means that celastrol enhanced doxorubicin‐mediated apoptosis in Saos‐2 cells.

### 3.4. Flow Cytometry Confirms Enhanced Apoptosis by Combination Treatment

**Figure 4:**
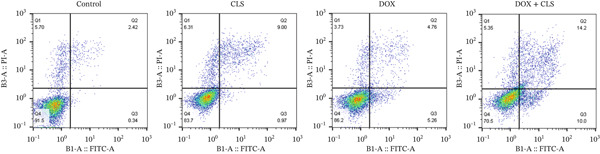
Flow cytometry analysis of apoptosis in Saos‐2 cells treated with celastrol and doxorubicin.

To further validate the proapoptotic effects observed through ELISA and molecular analyses, we performed flow cytometry using Annexin V‐FITC/PI double staining. As illustrated in Figure 4, control Saos‐2 cells showed minimal apoptosis, with only 0.34% in early apoptosis and 2.42% in late apoptosis, yielding a total apoptotic rate of 2.6%. Celastrol (4 *μ*M) monotherapy showed moderate effects with 0.97% early apoptotic cells and 9.0% late apoptotic cells (total apoptosis: 9.97%). Treatment with DOX (10 *μ*M) alone yielded results comparable with those with 5.26% early apoptotic cells and 4.76% late apoptotic cells (total apoptosis: 10.02%).

Remarkably, the combination of DOX and celastrol demonstrated synergistic proapoptotic effects, significantly increasing both early (10.0%) and late (14.2%) apoptotic cell populations, resulting in a total apoptotic rate of 24.2%. This represented a statistically significant increase compared with DOX alone and celastrol alone (*p* < 0.05), confirming the synergistic interaction between these agents. The necrotic cell population remained minimal across all treatment groups (< 6%), indicating that cell death occurred primarily by apoptosis rather than necrosis. These flow cytometry findings corroborate our ELISA results and molecular analyses, providing robust evidence that celastrol potentiates doxorubicin‐mediated apoptosis in Saos‐2 osteosarcoma cells.

## 4. Discussion

Our study’s results revealed that the combination of doxorubicin and celastrol could induce apoptosis in Saos‐2 cells by upregulating p53 and targeting key apoptosis mediators, including Bax, Bcl‐2, Caspase‐3, and cytochrome‐c [14]. During the development of innovative cancer treatments, natural products have been extensively studied for their ability to inhibit cell proliferation, induce apoptosis, and thus prevent cancer progression [15, 16]. Celastrol is among the most novel natural compounds with anticancer effects. Based on epidemiological studies, intake of natural compounds can effectively reduce the risk of certain human malignancies, including osteosarcoma [17]. Celastrol’s beneficial effects in cancer cells are due to its ability to suppress cell growth by targeting cell cycle regulatory proteins and arresting the cell cycle [18].

Wild‐type p53 is a short half‐life transcription factor with tight regulation during the cell cycle [19, 20]. The p53 protein levels are elevated in the majority of invasive breast cancer cells. These cells show higher levels of death resistance than normal cells [21, 22]. It has been demonstrated that some polyphenols, as well as curcumin, ursolic acid, and elagic acid, can induce apoptosis in certain tumor types by activating or up‐regulating wild‐type p53 [23, 24]. P53 induces apoptosis in cells with degraded DNA by regulating the Bax and Bcl‐2 family [22]. In the mitochondria, p53 induces oligomerization of Bax and Bak, which interact with protective Bcl‐2 and Bcl‐XL, antagonizing their antiapoptotic effects. It also forms a complex with cyclophilin D, contributing to mitochondrial structural disruption [19, 25]. Studies in this regard revealed that celastrol induced apoptosis via both intrinsic and extrinsic pathways, specifically mitochondrial and FAS (CD95) pathways, respectively. In the intrinsic pathway, celastrol treatment modulates caspase‐dependent anti‐/proapoptotic proteins by enhancing mitochondrial membrane potential and facilitating cytochrome c (Cyt‐c) release. This perspective aligns with our findings on celastrol’s anti‐Bcl‐2 activity. Bcl‐2 is located principally on the outer layer of mitochondria, and its upregulation prevents cells from undergoing apoptosis. Celastrol downregulates Bcl‐2 in carcinoma cells by reversing its inhibitory effect on the release of Cyt‐c from mitochondria into the cytosol, thereby initiating apoptosis, not to mention that cytosolic Cyt‐c is crucial for the beginning of the apoptotic program [26]. In the extrinsic pathway, the expression of FAS and FASL (FAS ligand) in cancer cells is modulated by celastrol, resulting in apoptosis. Additionally, celastrol treatment of cancer cells, compared with normal cells, enhances ROS and induces apoptosis, indicating that the ROS‐mediated mitochondrial caspase‐independent pathway is central to celastrol‐mediated apoptosis [18]. Celastrol, in combination with various chemotherapeutics, was reported to exert a synergistic effect and enhance therapeutic efficacy. For example, celastrol, in combination with lapatinib, produced strong synergy in the suppression of MDA‐MB‐453 cell proliferation and induction of apoptosis [27]. In another study by Li et al., the combination of apatinib and celastrol significantly potentiated Caspase‐3 and Bax expression and induced apoptosis in human hepatoma Hep3B cells [27]. Celastrol was also described to enhance the anti‐liver cancer activity of sorafenib. Celastrol enhanced the antitumor activity of sorafenib in hepatocellular carcinoma tumor cells by suppressing the AKT pathway and VEGF autocrine system. It also enhanced sorafenib’s growth inhibition and apoptosis induction in cancer cells, both in vitro and in vivo, and reduced the required sorafenib dosage [28].

Additionally, celastrol, when combined with epidermal growth factor receptor tyrosine kinase inhibitors, significantly suppressed the cell invasion of lung cancer cells with a T790M mutation by inhibiting the EGFR pathway [29]. Researchers found that combining ABT‐737 and celastrol synergistically suppressed HCC cell proliferation and induced apoptosis, accompanied by the activation of the caspase cascade and the release of cytochrome c from mitochondria [30]. As in previous studies, we reported that celastrol increased doxorubicin‐mediated apoptosis by downregulating Bcl‐2 expression and upregulating p53, cytochrome c, Bax, and caspase‐3 in osteosarcoma Saos‐2 cells [13].

Our flow cytometry analysis using Annexin V‐FITC/PI double‐staining provided complementary confirmation of the enhanced apoptotic response. The detection of phosphatidylserine externalization (Annexin V binding) on the outer membrane leaflet is a hallmark of early apoptotic events. At the same time, PI incorporation indicates loss of membrane integrity, a characteristic of late apoptosis. The combination treatment resulted in 24.2% total apoptosis, nearly double that of single‐agent therapies, with a predominant early apoptotic population suggesting active caspase‐mediated programmed cell death rather than passive necrosis. This temporal progression from early to late apoptosis, combined with minimal necrotic populations, supports the conclusion that celastrol‐enhanced DOX cytotoxicity operates primarily through controlled apoptotic mechanisms. These findings align with previous reports demonstrating that combination strategies targeting multiple apoptotic pathways can overcome resistance mechanisms and achieve superior therapeutic outcomes [30].

While our study provides robust evidence of apoptosis through multiple complementary approaches—including quantitative DNA fragmentation analysis and flow cytometry‐based methods (such as Annexin V/PI double staining), future studies employing imaging‐based approaches, such as confocal microscopy, can visualize the subcellular localization of apoptotic proteins and morphological changes. Additionally, detection of cleaved‐caspase‐3 by immunofluorescence or Western blot could complement our functional caspase‐3 activity data. However, we believe that our current comprehensive approach—integrating specific DNA fragmentation quantification, comprehensive molecular analysis of the apoptotic pathway, and direct measurement of caspase‐3 enzymatic activity—provides sufficient and rigorous evidence for our conclusions regarding the proapoptotic mechanisms of celastrol‐enhanced doxorubicin treatment in osteosarcoma cells. Also, future studies employing long‐term clonogenic survival assays (colony formation) would provide additional insights into the sustained antiproliferative effects and potential for complete eradication of cancer cell populations.

In conclusion, treatment with celastrol in combination with doxorubicin may produce strong synergy in suppressing Saos‐2 osteosarcoma cell proliferation and inducing apoptosis. These data create new insights into the antiproliferative properties of celastrol, which can be used as a complementary treatment alongside the principal therapy for osteosarcoma.

## Funding

This paper is the outcome of the MSc degree in the Clinical Biochemistry thesis (Thesis no. 71212) approved by Tabriz University of Medical Sciences.

## Ethics Statement

This study involved only in vitro experiments using established cell lines and did not involve animal or human subjects. All experimental procedures were applied following approval from the Ethics Committee of Tabriz University of Medical Sciences (IR.TBZMED.VCR.REC.1402.121).

## Conflicts of Interest

The authors declare no conflicts of interest.

## Supporting information


**Supporting Information** Additional supporting information can be found online in the Supporting Information section. Figure S1: Time‐dependent cytotoxic effects of celastrol on Saos‐2 osteosarcoma cells. Cell viability was assessed using the MTT assay at 0, 12, 24, 48, and 72 h following treatment with 4 *μ*M celastrol. The data demonstrate a progressive reduction in cell viability over time, confirming the time‐dependent antiproliferative effect of celastrol.

## Data Availability

The data supporting this study’s findings are available on request from the corresponding author.
