# Supplementary material for: Celastrol Enhanced Doxorubicin‐Mediated Apoptosis in Saos‐2 Osteosarcoma Cells
Source: Biomed Res Int. 2026 May 18;2026:8836764. doi: 10.1155/bmri/8836764 (PMC13184160; doi:10.1155/bmri/8836764)
Supplement: Supplementary file 1 — Supporting Information Additional supporting information can be found online in the Supporting Information section. Figure S1: Time‐dependent cytotoxic effects of celastrol on Saos‐2 osteosarcoma cells. Cell viability was assessed using the MTT assay at 0, 12, 24, 48, and 72 h following treatment with 4 μM celastrol. The data demonstrate a progressive reduction in cell viability over time, confirming the time‐dependent antiproliferative effect of celastrol. [file BMRI-2026-8836764-s001.docx]

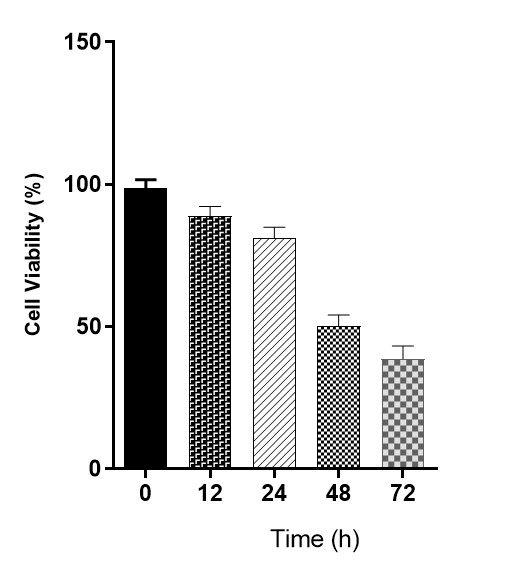


**Supplementary Figure S1.** Time-course analysis of celastrol-induced cytotoxicity in Saos-2 osteosarcoma cells measured by MTT assay at 0, 12, 24, 48, and 72 hours. The data demonstrate the time-dependent reduction in cell viability following treatment with 4 μM celastrol.
